# Influence of Solvent-Dependent Morphology on Molecular Doping and Charge Transport in Conductive Thiophene Polymer

**DOI:** 10.3390/ma15093293

**Published:** 2022-05-04

**Authors:** Haoyu Chai, Hui Li, Fei Zhong, Zhen Xu, Shengqiang Bai, Lidong Chen

**Affiliations:** 1 School of Materials Science and Engineering, Jingdezhen Ceramic University, Jingdezhen 333403, China; 1920024009@stu.jci.edu.cn; 2 State Key Laboratory of High Performance Ceramics and Superfine Microstructure, Shanghai Institute of Ceramics, Chinese Academy of Sciences, Shanghai 200050, China; zhongfei21@mails.ucas.ac.cn (F.Z.); xuzhen20@mails.ucas.ac.cn (Z.X.); bsq@mail.sic.ac.cn (S.B.); cld@mail.sic.ac.cn (L.C.); 3 Center of Materials Science and Optoelectronics Engineering, University of Chinese Academy of Sciences, Beijing 100049, China

**Keywords:** conductive polymer, chemical doping, electrical conductivity, carrier mobility

## Abstract

The utility of a solvent is one of the key factors that impacts resultant film morphology. However, the effect of solvent-dependent morphology on the doping process and electrical conductivity has not been adequately elucidated. In this work, we compared the morphology of chloroform- and chlorobenzene-processed thiophene polymer films and investigated how the choice of solvent influences film morphology, doping level, charge transport properties, and thus electrical conductivity. It was found that the film drop-casted from chloroform exhibits better crystallinity than that drop-casted from chlorobenzene. The crystallinity has negligible impact on the doping level but significant impact on charge transport properties. As a result, the chloroform-processed film shows a higher electrical conductivity of up to 408 S cm^−1^ due to a high carrier mobility related to the continuously crystalline domains in film. This finding indicates that the choice of solvent for preparation of film, which strongly correlated with molecular orientation, is a new strategy to optimize the electrical conductivity of doped polymers.

## 1. Introduction

Molecular doping has been a promising way to control the electrical properties of polymer semiconductors [1,2]. The doped conjugated polymers are widely used as the active layers in the organic field-effect transistor, organic light-emitting diode, and organic thermoelectric devices [3,4,5,6,7]. Molecular doping will improve the electrical conductivity (*σ*) by increasing free carriers along polymer chains via charge transfer between polymer and dopant. To achieve a high *σ*, an extremely high doping concentration is usually required to obtain a high carrier concentration (*n*) [8,9]. However, the addition of large amounts of dopants results in a disruption of the polymer film morphology and induces a drop in carrier mobility (*μ*) [10], which leads to a low *σ* according to the relationship of *σ* = *n*e*μ*. [8] Therefore, it is still challenging to effectively dope polymers without restraining the charge transport in polymer film. There have been great efforts recently to overcome film quality issues of heavily doped conjugated polymers. For example, several doping methods are developed in which the dopants are applied to a pre-casted polymer film, including vapor-phase deposition [11,12], immersion doping [13,14], sequential doping [15,16], etc. Other attention has been given to the modification of chemical structures of polymers to leave dopants in side chains without disturbing the packing of polymer backbones [17,18]. For instance, the introduction of π-conjugated subunits as a spacer can reduce the number of side chains and improve the accommodation of dopants into films [19]. Additionally, polythiophenes with oligo(ethylene oxide) side chains show high electrical conductivity of up to 100 S cm^−1^ and is attributed to the good miscibility between polar side chains and dopants [20,21]. It is accepted that the crystalline film of pristine polymers is beneficial for achieving good charge transport properties. The crystalline behavior of polymers is strongly related to the properties of the solvent for dissolving the given polymer [22,23]. However, how the solvent affects the solid-state orientation of pristine film, and therefore the molecular doping and charge transport in doped film, has not been sufficiently elucidated.

Herein, we investigate the influence of solvent for dissolving the polymer on the crystallinity of pristine polymer films, doping efficiency and charge transport by employing a thiophene polymer, PODTT-4T, drop-casted from chloroform (CF) and chlorobenzene (CB) solution, respectively. It is found that the pristine film prepared from CF solution (PODTT-4T_CF_) shows a preferentially edge-on orientation and a highly ordered microstructure while the film prepared from CB solution (PODTT-4T_CB_) exhibits a lower crystallinity with both edge-on and face-on orientations inside. PODTT-4T_CF_ film shows a much higher *σ* of up to 408 S cm^−1^ compared to that of the PODTT-4T_CB_ (280 S cm^−1^) although these two films have a similar doping level if doped by FeCl_3_. The higher *σ* of doped PODTT-4T_CF_ can be ascribed to the higher carrier mobility due to larger crystalline domains upon doping compared to the PODTT-4T_CB_ film. We propose that the choice of solvent to dissolve PODTT-4T determines the polymer crystalline behavior in the solid state, which prefers to dominate the transport property of free carriers rather than the doping efficiency.

## 2. Synthesis and Characterization of Polymer

**Scheme 1:**
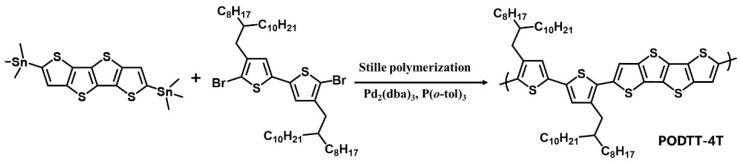
The synthetic route to the polymer PODTT-4T.

Polymer PODTT-4T was synthesized by Stille coupling reaction [24], copolymerizing 5,5’-dibromo-4,4’-bis(2-octyldodecyl)-2,2’-bithiophene and 2,6-bis(trimethylstannyl)thieno [2’,3’:4,5]thieno[3,2-*b*]thieno[2,3-*d*]thiophene (4T) (Scheme 1). The large conjugated unit is used to allow good planarity, promoting the charge delocalization. The long and branched alkyl chains ensure the solubility of copolymers in common organic solvents, such as CF and CB. The synthesis details and basic properties of the polymer are given in the supporting information. PODTT-4T shows a high number-average molecular weight (*M*_n_) of 22.2 kDa with a polydispersity index (PDI) of 2.08 (Figure S1). It exhibits excellent thermal stability with a decomposition temperature (5% weight loss) of about 395 ℃ measured by thermogravimetric analysis (TGA) (Figure S2). Differential scanning calorimetry (DSC) analysis shows a melting transition peak at 296 °C and a crystallization peak at 271 °C during the heating and cooling cycle (Figure S3), respectively, which suggests the existence of crystalline phases in the polymer [25]. PODTT-4T shows the highest occupied molecular orbit (HOMO) level around −5.26 eV by employing the cyclic voltammetry (CV) (Figure S4), which is beneficial for p-doping by FeCl_3_ [26]. The pristine films were prepared by dissolving the polymer into CF or CB with a concentration of 5 mg mL^−1^. The polymer solution was drop-casted on the glass substrate and dried at ambient temperature. Then, the films were annealed at 160 °C for 30 min in the glovebox. The doped films were prepared by immersing the annealed films into FeCl_3_ solution (in acetonitrile, 5 mg mL^−1^) for 10 min.

## 3. Results

### 3.1. The Doping Level of Films Prepared from Two Solvents

**Figure 1:**
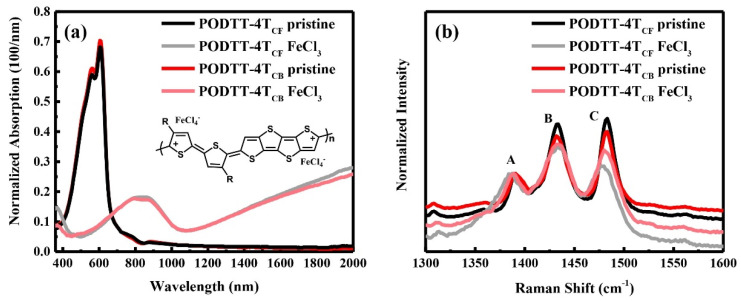
(**a**) The thickness-normalized absorption spectra; (**b**) Raman spectra of pristine and doped films drop casted from CF and CB, respectively. The Raman intensity is normalized on the band peaked at mode A.

The doping behavior of PODTT-4T_CF_ and PODTT-4T_CB_ films is characterized by using UV-vis-NIR absorption spectra. It is observed that pristine PODTT-4T_CF_ and PODTT-4T_CB_ films show a similar absorption spectrum (Figure 1a). The maximum peak appears at 605 nm and no obvious absorption is observed over 700 nm. In addition, there is no obvious peak shift or absorption intensity change between these two films although the different solvents usually induce the wavelength shift of shoulder peaks due to the change of backbone interactions [27]. This indicates PODTT-4T chains dissolved in CF and CB probably adopt a similar π-π stacking distance in the solid state. Upon doping with FeCl_3_, the intensity of the neutral absorption peak of PODTT-4T_CF_ and PODTT-4T_CB_ films is significantly bleached. Meanwhile, the absorption band ascribed to the (bi)polarons appears above 800 nm in the near-infrared region. In order to compare the doping levels of the two films, two absorption bands—neutral absorption band_Ⅰ_ (380–710 nm) and (bi)polaron absorption band_Ⅱ_ (710–2000 nm)—are assigned. The ratio (*R*) of the integral area of these two absorption bands (band_II_/band_I_) is calculated (Table S1). It is found that the doped PODTT-4T_CF_ film and PODTT-4T_CB_ film have a similar *R* value suggesting similar doping levels in the two films [21]. This indicates that CF and CB have a negligible effect on the polymer doping level under the same conditions. Raman spectroscopy (Figure 1b) is further employed to characterize and analyze the vibrational mode of PODTT-4T_CF_ and PODTT-4T_CB_ upon doping. All intensities are normalized at the vibrational peak of mode A, which corresponds to the C=C symmetric stretching vibration on the 4T core. Mode B and mode C correspond to the C=C stretching vibration and the C=C/C-C stretching/shrinking on the thiophene ring, respectively [28]. These vibrational modes are sensitive to the structural order and π-electron delocalization [29]. The positions of mode B and mode C of the pristine PODTT-4T_CF_ film and PODTT-4T_CB_ film are almost the same while mode A of PODTT-4T_CB_ is wider than that of PODTT-4T_CF_, indicating more disordered structures existing in the former film. Upon doping with FeCl_3_, both mode A and mode C shift to much lower wavenumbers, suggesting that the benzenoid structures along the conjugated backbone are transformed into quinoid structures. It should be noted that the shift of mode C of PODTT-4T_CF_ is more significant (Table S2) and mode A is much wider compared to those of PODTT-4T_CB_ film, which means the dopants have a significant influence on the molecular packing of polymer chains in PODTT-4T_CF_ film. These results demonstrate that the doping level is not dependent on the molecular orientation in PODTT-4T film.

### 3.2. The Electrical Conductivity of Doped Films

**Figure 2:**
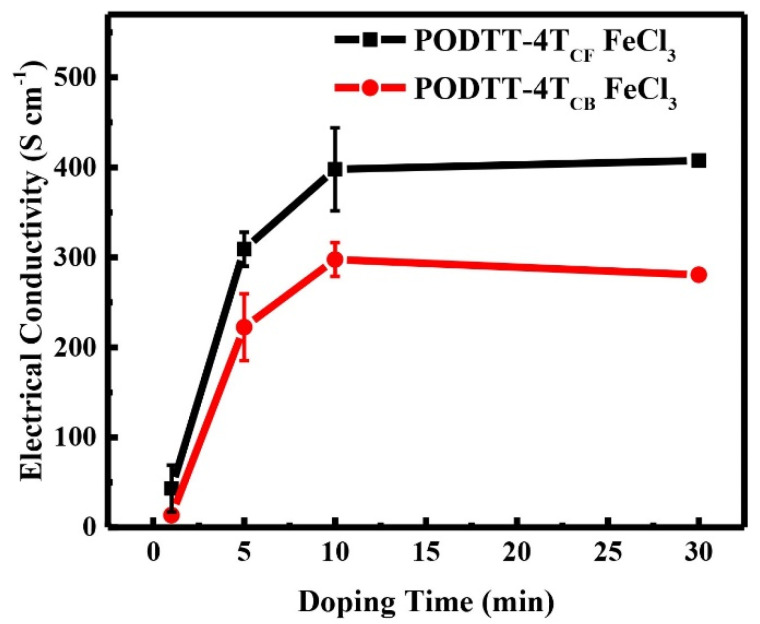
Electrical conductivities of PODTT-4T_CF_ (black line) and PODTT-4T_CB_ (red line) doped by FeCl_3_ as a function of the doping time.

To investigate how the solvents impact the electrical properties, the electrical conductivity of PODTT-4T_CF_ and PODTT-4T_CB_ films doped by FeCl_3_ was tested by four-probe measurement. As shown in Figure 2, the electrical conductivities of the two films dramatically increase with increasing the immersing time in the range of 10 min. As a result, the *σ* tend to be saturated. The *σ* of doped PODTT-4T_CF_ film is higher than that of doped PODTT-4T_CB_ film if doping time is greater than 10 min. The highest *σ* of PODTT-4T_CF_ is up to 408 S cm^−1^, which is 1.5 times higher than that of PODTT-4T_CB_ (280 S cm^−1^). In addition, the Seebeck coefficient (*S*, the potential difference arises from per unit temperature difference) of the doped PODTT-4T_CF_ film is slightly higher than that of the PODTT-4T_CB_ film (Figure S5a). As a result, the power factor (*S*^2^*σ*) of the doped PODTT-4T_CF_ film is two times higher than that of the PODTT-4T_CB_ film at a doping time of 10 min, indicating the potential thermoelectric application of this polymer. Considering a similar doping level observed in two-doped films, the higher TE performance could probably be attributed to the higher mobility in PODTT-4T_CF_ film. To further support our hypothesis, Hall effect measurements were employed to investigate the effect of solvents on carrier concentrations and mobilities in doped polymers at doping times of 10 min (Figure S6). The results show that the mobility of the doped PODTT-4T_CF_ film and PODTT-4T_CB_ film is 0.96 cm^2^ V^−1^ s^−1^ and 0.76 cm^2^ V^−1^ s^−1^, respectively. Meanwhile, the carrier concentration of the doped PODTT-4T_CF_ film and PODTT-4T_CB_ film is 2.74 × 10^21^ cm^−3^ and 2.62 × 10^21^ cm^−3^, respectively (Table S3). These data indicate that both films show higher mobilities than that of a typical thiophene polymer, such as FeCl_3_-doped P3HT [13], due to a large and delocalized backbone of PODTT-4T. In addition, the PODTT-4T_CF_ film exhibits a 1.3-fold higher carrier mobility with slightly more free carriers compared to PODTT-4T_CB_ film, affirming the importance of solvent selection for electrical conductivity optimization.

### 3.3. Microstructural Characterization

**Figure 3:**
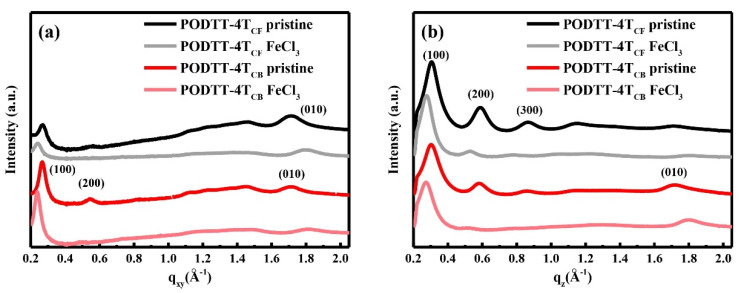
(**a**) GIWAXS line cuts of in-plane scattering profiles; (**b**) GIWAXS line cuts of out-of-plane scattering profiles of pristine and doped films drop-cast from CB and CB, respectively.

To further understand the relationship between solvent-dependent morphology and charge transport properties, grazing-incidence wide-angle X-ray scattering (GIWAXS) was performed (Figure 3 and Figure S7). Pristine PODTT-4T_CF_ film shows intense (*h*00) multi-order diffraction peaks in the out-of-plane direction (along the q_z_ direction) and a (010) diffraction peak at q_xy_ = 1.6–1.8 Å^−1^ generated by π-π stacking in the in-plane direction. That indicates a dominantly edge-on orientation existing in PODTT-4T_CF_ film [30,31]. In contrast, pristine PODTT-4T_CB_ film shows weak and broad Debye rings of (*h*00) peaks with a (010) diffraction peak appearing both in the out-of-plane and in-plane directions, meaning that both edge-on and face-on orientation exists in the PODTT-4T_CB_ film. Since CF and CB have different boiling points, the difference of molecular orientations from two solvents can be attributed to the evaporation rate of solvent during the film preparation. In addition, the face-on orientation is thermodynamically stable packing for the polymer. The lamellar distance and π-π stacking distance of the PODTT-4T_CF_ film are 20.26 Å and 3.67 Å, while the distances are 20.93 Å and 3.67 Å for PODTT-4T_CB_ film, respectively. (Table S4). These results demonstrate that the utility of solvent mainly affects the packing orientation in pristine films and has a negligible effect on the lamellar distance and π-π stacking distance. Upon doping by FeCl_3_, the lamellar stacking distance of PODTT-4T_CF_ film is increased to 22.43 Å (Δ*d*_100_ = 2.17 Å), while the π-π stacking distance decreases to 3.50 Å (Δ*d*_010_ = −0.17 Å). For the PODTT-4T_CB_ film, the lamellar packing distance is increased to 23.26 Å (Δ*d*_100_ = 2.33 Å) while the π-π stacking distance also decreases from 3.67 to 3.50 Å (Δ*d*_010_ = −0.17 Å). These results indicate that the dopants prefer to intercalate into the lamellar alkyl side-chain region without disturbing the intense π-π stacking attributed to the electronic coupling between the conjugated backbone. Moreover, the molecular orientations of pristine films are still maintained upon doping. The size of the crystalline grain of pristine and doped films is compared by extracting the data of the full-width at half-maximum (FWHM) of (100) peaks according to the Scherrer equation [32,33]. It is observed that the FWHM of pristine PODTT-4T_CF_ film (0.072 Å^−1^) is slightly smaller than that of pristine PODTT-4T_CB_ film (0.078 Å^−1^), indicating larger crystalline domains in the pristine PODTT-4T_CF_ film. Upon doping, the crystalline domains of doped films are significantly increased. In addition, the crystalline size of doped PODTT-4T_CF_ film is still much larger than that of doped PODTT-4T_CB_ film.

**Figure 4:**
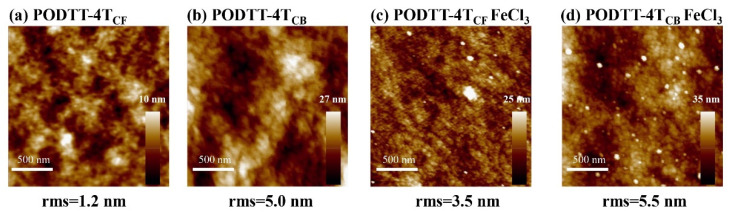
AFM height images of (**a**) pristine PODTT-4T_CF_ film, (**b**) pristine PODTT-4T_CB_ film, (**c**) doped PODTT-4T_CF_ film, and (**d**) doped PODTT-4T_CB_ film.

The microscopic morphology of the pristine and doped films is further characterized by atomic force microscopy (AFM) (Figure 4). Both the pristine PODTT-4T_CF_ and pristine PODTT-4T_CB_ films are smooth with an average roughness of 1.2 nm and 5.0 nm, respectively. Pristine PODTT-4T_CF_ shows a fibrous zone while PODTT-4T_CB_ film looks amorphous, which is consistent with the GIWAXS results. The surface roughness of doped films is slightly increased, which is in agreement with the enlarged crystalline domains observed in GIWAXS. For the doped PODTT-4T_CF_ film, the self-aggregation of dopants is not obviously observed at the surface compared to that of PODTT-4T CB film, suggesting better miscibility of FeCl_3_ with the former film. The GIWAXS information and AFM results support the fact that the ordered molecular packing and higher crystallinity in PODTT-4T_CF_ film are responsible for its higher mobility compared to that of PODTT-4T_CB_ film. The use of chloroform solvent can promote polymer chains to form more ordered packing and larger crystalline domains.

**Figure 5:**
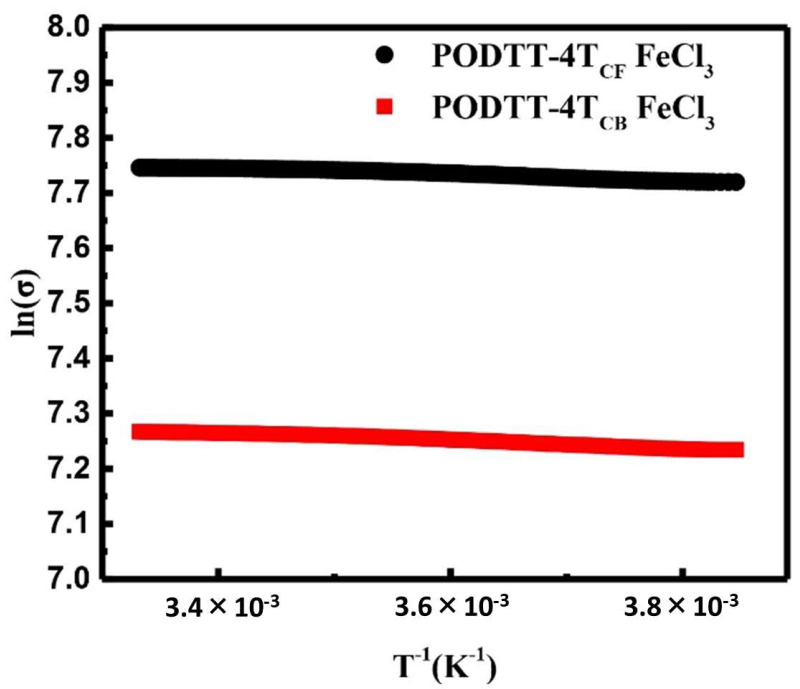
Temperature-dependent electrical conductivity of doped PODTT-4T_CF_ and PODTT-4T_CB_ films (the unit of *σ* is S cm^−1^). All doped films were immersed in FeCl_3_ solution for 10 min.

To further elucidate the carrier transport mechanism in the doped PODTT-4T_CF_ and PODTT-4T_CB_ films, the temperature-dependent *σ* was measured. It has been found that the *σ* increases with increased temperature (Figure 5), indicating thermal activated transport properties in doped films [34,35]. The transport activation energy (*E*_a_) of the doped PODTT-4T_CF_ film and PODTT-4T_CB_ film can be calculated according to the equation of *σ*=*σ*_0_exp(−*E*_a_/*k*_B_*T*), where *σ*_0_ is the pre-exponential conductivity and *k*_B_ is Boltzmann constant [35]. It has been found that the *E*_a_ of the doped PODTT-4T_CF_ film is 5.2 meV, which is lower than that of doped PODTT-4T_CB_ films (*E*_a_ = 8.0 meV). The low *E*_a_ suggests a low transport barrier exists, and thus more polaronic states can contribute to electrical conductivity due to the continuous carrier transport channels in the doped PODTT-4T_CF_ film [36]. Therefore, the film prepared from CF solution allows highly ordered and crystalline domains, resulting in strong coupling between doped polymer chains and thus a high carrier mobility.

## 4. Conclusions

In this work, the effect of solvent on morphology, doping process and electrical conductivity of thiophene polymers has been investigated. It has been found that the molecular orientation in films is strongly related to the solvent used for dissolving the polymer, which is dominated by the evaporation rate of solvent during film formation. The films prepared from chloroform (PODTT-4T_CF_) mainly have an edge-on orientation, while the films prepared from chlorobenzene (PODTT-4T_CB_) show both edge-on and face-on orientations. PODTT-4T_CF_ film exhibits better crystallinity than that of the chlorobenzene-processed film. The crystallinity has significant influence on charge transport properties rather than the doping level. As a result, FeCl_3_-doped PODTT-4T_CF_ film shows a higher electrical conductivity of up to 408 S cm^−1^ and is attributed to a high carrier mobility correlated to the continuous and largely crystalline domains in film compared to that of chlorobenzene-processed film. Our findings show that the selection of solvents for the preparation of film is a promising strategy to optimize the electrical conductivity of doped polymers.

## Data Availability

The data that supports the findings of this study are available from the corresponding author upon reasonable request.

## Supplementary Materials

The following supporting information can be downloaded at: https://www.mdpi.com/article/10.3390/ma15093293/s1, Figure S1: GPC trace of PODTT-4T; Figure S2: TGA curve of PODTT-4T; Figure S3: DSC scan curves of PODTT-4T; Figure S4: Cyclic voltammetry profiles of PODTT-4T; Figure S5: Thermoelectric performance; Figure S6: Hall effect measurement; Figure S7: 2D-GIWAXS images; Table S1: The ratio of the integrated area between (bi)polaron band and the neutral band; Table S2: Raman spectroscopy; Table S3: The mobility and carrier concentration; Table S4: Data of 2D-GIWAXS.
