# Supplementary material for: Influence of Solvent-Dependent Morphology on Molecular Doping and Charge Transport in Conductive Thiophene Polymer
Source: Materials (Basel). 2022 May 4;15(9):3293. doi: 10.3390/ma15093293 (PMC9105990; doi:10.3390/ma15093293)
Supplement: Supplementary file 1 [file materials-15-03293-s001.zip › materials-1696309-supplementary.pdf]

materials MDPI

Supporting Information

Influence of Solvent-Dependent Morphology on Molecular Doping and Charge Transport in Conductive Thiophene-Polymer

Haoyu Chai [1,2], Hui Li [2,*], Fei Zhong [2], Zhen Xu [2], Shengqiang Bai [2,3] and Lidong Chen [2,3]

[1] School of Materials Science and Engineering, Jingdezhen Ceramic University, Jingdezhen 333403, China; 1920024009@stu.jci.edu.cn
[2] State Key Laboratory of High Performance Ceramics and Superfine Microstructure, Shanghai Institute of Ceramics, Chinese Academy of Sciences, Shanghai 200050, China; zhongfei21@mails.ucas.ac.cn (F.Z.); xuzhen20@mails.ucas.ac.cn (Z.X.); bsq@mail.sic.ac.cn (S.B.); cld@mail.sic.ac.cn (L.C.)
[3] Center of Materials Science and Optoelectronics Engineering, University of Chinese Academy of Sciences, Beijing 100049, China
* Correspondence: lihui889@mail.sic.ac.cn

Synthesis of PODTT-4T

The synthesis of polymer was carried out by Stille coupling under an N_2 atmosphere according to the literature [1]. 2,6-Bis(trimethylstannyl)thieno[2',3':4,5]thieno[3,2-*b*]thieno[2,3-*d*]thiophene (97.7 mg, 0.169 mmol) was added into a dry thick-walled pressure bottle, which is moderately heated with a heat gun to remove moisture under nitrogen. 5,5'-Dibromo-4,4'-bis(2-octyldodecyl)-2,2'-bithiophene (150 mg, 0.169 mmol) was dissolved in chlorobenzene (4 mL) and then added into the bottle. P(*o*-tol)$_3$ (6.2 mg, 0.02 mmol) and $Pd_2(dba)_3$ were added. The reaction mixture was degassed for 10 min and afterward sealed. The bottle was then heated at 120 °C and stirred for 48 hours. After cooling to room temperature, the mixture was precipitated into methanol. The filtered products were washed with methanol, acetone, hexane, and finally chloroform by Soxhlet extraction. The final product was dissolved in chloroform again, then the solvent was concentrated and dropped in methanol. The filtered precipitate was dried at 50 °C for 12 hours in the glovebox to obtain the desired polymer (219 mg, 88%). GPC: M_n = 22.2 kDa, PDI = 2.08.

Gel permeation Chromatography

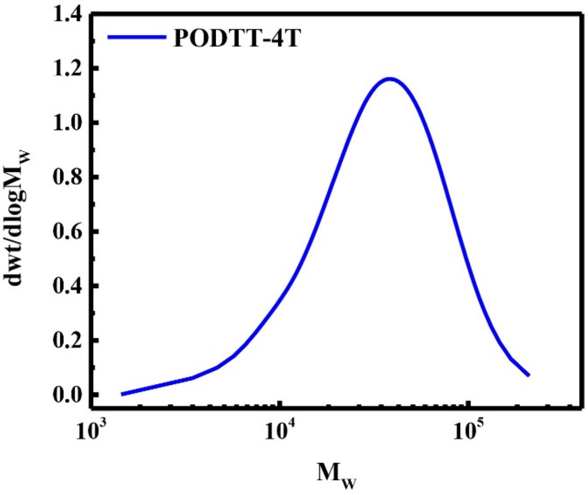

Figure S1. GPC trace of PODTT-4T with 1,2,4-tricholorobenzene as eluent at 150 °C.

Thermal Properties

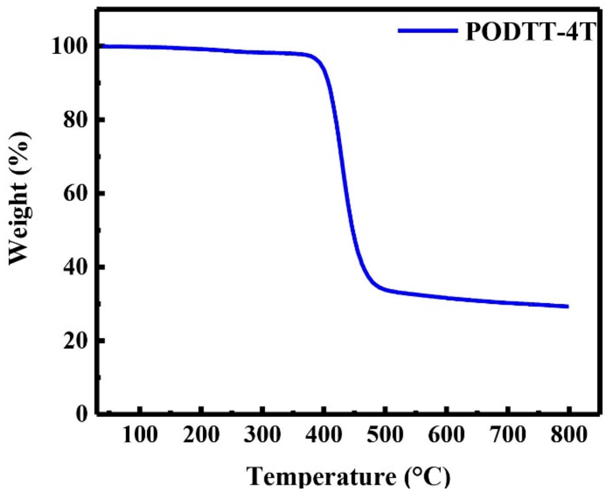

Figure S2. TGA curve of PODTT-4T under nitrogen.

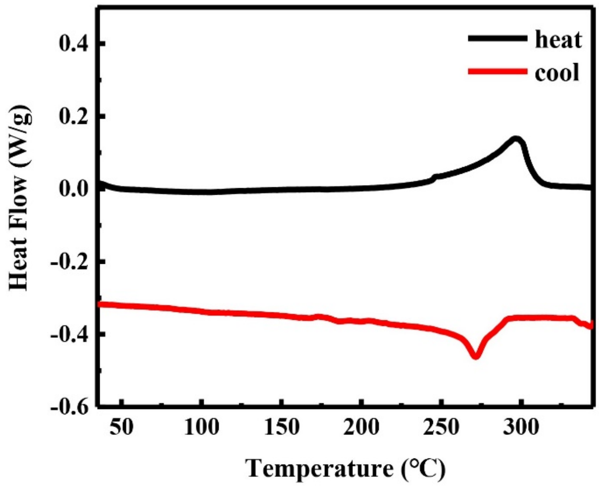

Figure S3. DSC scan curves of PODTT-4T with a scan rate of 10 °C/min under nitrogen.

Cyclic Voltammetry

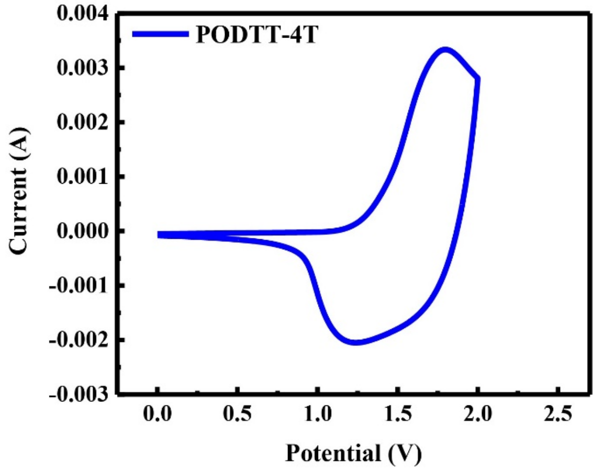

Figure S4. Cyclic voltammetry profiles of PODTT-4T. HOMO level can be calculated by HOMO = -e(E_{ox} + 3.99) (eV) (the redox potential of Fc/Fc^+ as external calibration is measured under the same conditions and it is located at 0.81 V to the Ag/AgCl electrode).

Cyclic voltammetry measurement was performed on a CH-Instruments 600E Electrochemical Workstation as previously reported [2]. A three-electrode setup was used with a platinum plate as working electrode, platinum wire as a counter electrode, and Ag/Ag^+ (fill solution: saturated KCl in CH_3CN) as a reference electrode. A 0.1 M solution of tetrabutylammonium hexafluorophosphate (Bu_4NPF_6) in anhydrous acetonitrile was used as a supporting electrolyte. For calibration, the redox potential of ferrocene/ferrocenium (Fc/Fc^+) was measured under the same conditions and it is located at 0.81 V to the Ag/AgCl electrode. It is assumed that the redox potential of Fc/Fc^+ has an absolute energy level of −4.80 eV to vacuum. The band gap of polymer can be extracted from the absorption spectra of polymer film ($E_g^{opt} = hc/\lambda_{onset}$). PODTT-4T$_{CF}$ and PODTT-4T$_{CB}$ films show the same optical band gap around 1.75 eV, and the E_{LUMO} is −3.51 eV calculated according to the equation of $E_{LUMO} = E_{HOMO} + E_g^{opt}$.

UV-Vis-NIR

Table S1. The ratio of the integrated area between (bi)polaron band and the neutral band for doped polymers (Band$_{I}$ and Band$_{II}$ mean the range of 380~710 nm and 710~2000 nm, respectively.).

Copyright: © 2022 by the authors. Licensee MDPI, Basel, Switzerland. This article is an open access article distributed under the terms and conditions of the Creative Commons Attribution (CC BY) license (https://creativecommons.org/licenses/by/4.0/).

**Film**	**Band$_{I}$ (380-710 nm)**	**Band$_{II}$ (710-2000 nm)**	**Ratio = Band$_{II}$/Band$_{I}$**
PODTT-4T$_{CF}$	111	27	
PODTT-4T$_{CF}$ $FeCl_3$	29	210	7.24
PODTT-4T$_{CB}$	115	25	
PODTT-4T$_{CB}$ $FeCl_3$	28	202	7.21

Raman

Table S2. The excitation wavelength selected for the Raman spectroscopy test is 532 nm, and the test range is 1300~1600 cm^{-1} (the number in square brackets means the increased shift compared with the pristine film).

Film	Mode A	Mode B	Mode C
PODTT-4T$_{CF}$	1390	1434	1483
PODTT-4T$_{CF}$ $FeCl_3$	1387[−3]	1434	1476[−7]
PODTT-4T$_{CB}$	1390	1432	1483
PODTT-4T$_{CB}$ $FeCl_3$	1389[−1]	1432	1480[−3]

TE Properties

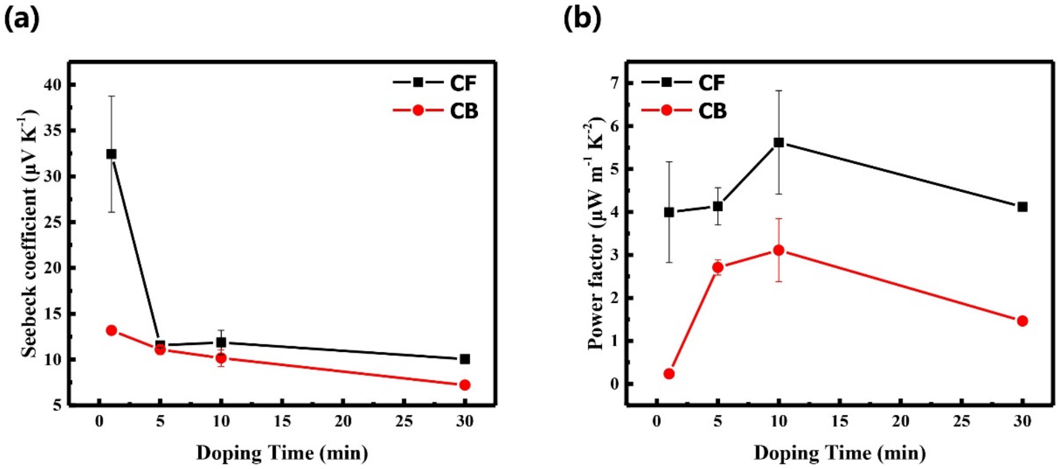

Figure S5. (**a**) Seebeck coefficient and (**b**) Power factor of doped PODTT-4T$_{CF}$ film (black) and PODTT-4T$_{CB}$ film (red). Film thickness was obtained by Bruker DektakXT contact profilometer.

The Seebeck coefficient (*S*) is measured by using the ZEM-5 TF Seebeck Coefficient/Electrical Resistance Measurement System (Advance Riko). At least three measurements of resistance and Seebeck coefficient were performed on each sample. The power factor was calculated by $S^2\sigma$.

Hall-effect Measurement

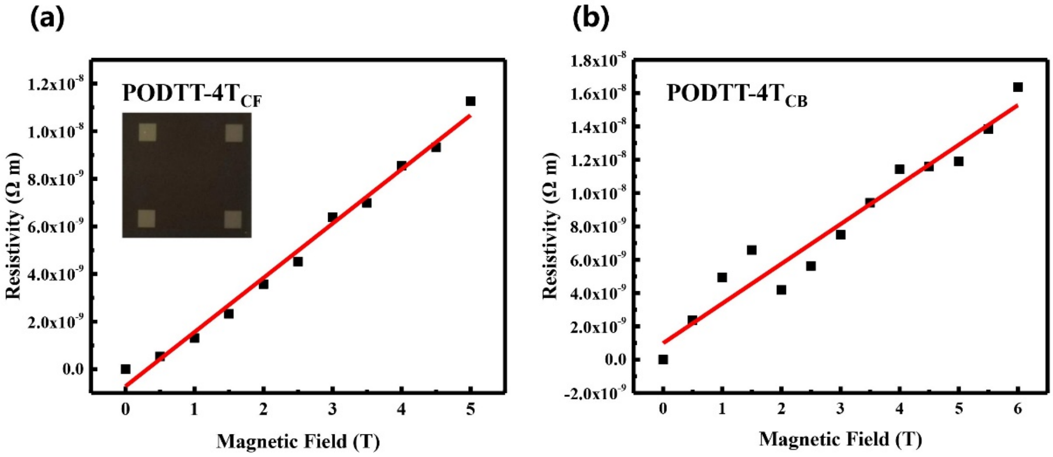

Figure S6. Resistivity as a function of the applied magnetic field for (a) PODTT-4T$_{CF}$ and (b) PODTT-4T$_{CB}$ films (red solid lines are fitted lines). The inset is the picture of sample for Hall-effect measurement.

Table S3. The mobility and carrier concentration of $FeCl_3$-doped PODTT-4T$_{CF}$ and PODTT-4T$_{CB}$ films were measured by AC-Hall measurement with the van der Pauw method.

Sample	n (10^{21} cm^{-3})	μ (cm^2 V^{-1} s^{-1})
PODTT-4T$_{CF}$ $FeCl_3$	2.74	0.96

PODTT-4T$_{CB}$ $FeCl_3$	2.62	0.76

Hall effect measurements were performed under He atmosphere using a Quantum Design PPMS with a custom system.[2] The preparation of the Hall effect measurement film is the same as that of the thermoelectric measurement film. Four Au electrodes with a thickness of 50 nm were deposited on the corners of the square film to reduce contact resistance. All films were analyzed using Vanderburgh geometry. It should be noted that we only focus on qualitative trends rather than the exact quantitative values for carrier mobility and carrier concentration.

GIWAXS

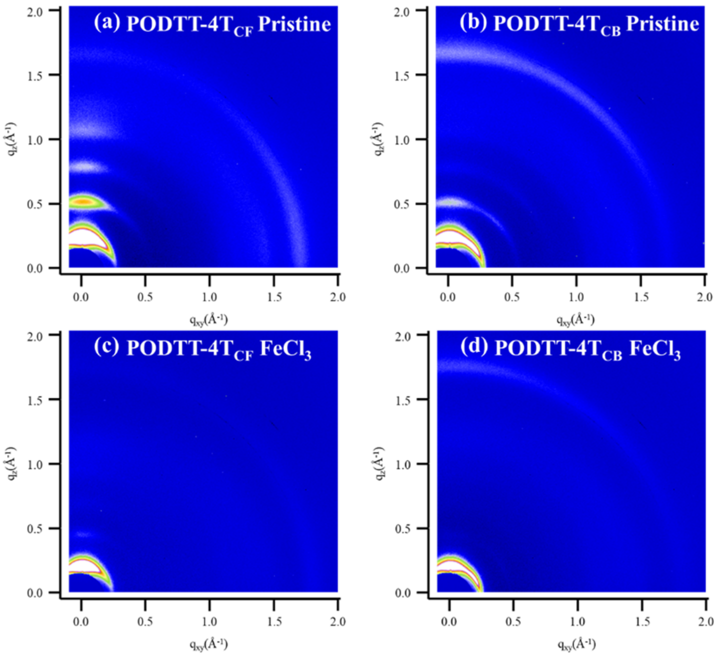

Figure S7. (**a**,**b**) 2D-GIWAXS images of pristine PODTT-4T$_{CF}$ and PODTT-4T$_{CB}$ films. (**c**,**d**) 2D-GIWAXS images of $FeCl_3$-doped films.

Grazing-Incidence Wide-Angle X-ray Scattering (GIWAXS) data were recorded at beamline BL14B1 of the Shanghai Synchrotron Radiation Facility (SSRF) at a wavelength of 1.24 Å.

Table S4. Lamellar (q_{100}) and π-π stacking (q_{010}) parameters of pristine and doped films of PODTT-4T$_{CF}$ and PODTT-4T$_{CB}$ (the number in square brackets means the increased distance compared with pristine film).

**Polymer**	**Out of Plane**					**In Plane**		
	**$q_{(100)}$ (Å^{-1})**	**d-spacing (Å)**	**$q_{(010)}$ (Å^{-1})**	**π-π stacking distance (Å)**	**FWHM (Å^{-1})**	**$q_{(100)}$ (Å^{-1})**	**$q_{(010)}$ (Å^{-1})**	**π-π stacking distance (Å)**
PODTT-4T$_{CF}$ pristine	0.31	20.26	-	-	0.072	0.27	1.71	3.67
PODTT-4T$_{CF}$ $FeCl_3$	0.28	22.43 [2.17]	-	-	0.053	0.24	1.80	3.50 [−0.17]

PODTT-4T$_{CB}$ pristine	0.30	20.93	1.72	3.65	0.079	0.27	1.71	3.67
PODTT-4T$_{CB}$ $FeCl_3$	0.27	23.26 [2.33]	1.80	3.49 [−0.16]	0.060	0.24	-	-

References

1. Li, H.; Song, J.; Xiao, J.; Wu, L.; Katz, H.E.; Chen, L. Synergistically Improved Molecular Doping and Carrier Mobility by Copolymerization of Donor–Acceptor and Donor–Donor Building Blocks for Thermoelectric Application. *Adv. Funct. Mater.* **2020**, *30*, 2004378. doi:10.1002/adfm.202004378.
2. Wu, L.; Li, H.; Chai, H.; Xu, Q.; Chen, Y.; Chen, L. Anion-Dependent Molecular Doping and Charge Transport in Ferric Salt-Doped P3HT for Thermoelectric Application. *ACS Appl. Electron. Mater.* **2021**, *3*, 1252–1259, doi:10.1021/acsaelm.0c01067.
